# Commentary: Two-sample Mendelian randomization: opportunities and challenges

**DOI:** 10.1093/ije/dyw127

**Published:** 2016-07-17

**Authors:** Debbie A Lawlor

**Affiliations:** MRC Integrative Epidemiology Unit at the University of Bristol and School of Social and Community Medicine, University of Bristol, Oakfield House, Oakfield Grove, Bristol BS8 2BN, UK.

## Introduction

In this volume of the *IJE* , Gao and colleagues explore the causal effect of adiposity on several cancers using two-sample Mendelian randomization (MR), and find some evidence that greater adult body mass index (BMI) causally reduces the risk of breast cancer while increasing ovarian, lung and colorectal cancer. ^1^ The authors conclude that the study provides ‘…additional understanding of the complex relationship between adiposity and cancer risks’.

Beyond the study findings themselves, this paper is interesting in its use of publicly available genome-wide association study (GWAS) summary data in a two-sample MR approach. Whereas MR has been increasingly used over the past decade since it was first proposed in the *IJE* , ^2^ two-sample MR is a relatively recent extension. ^3^ With the increasing availability of complete summary results from GWAS consortia that are easily accessible on the internet, the use of two-sample MR is likely to increase considerably over the next decade. ^4^ By complete summary data I mean results for all genetic loci with a trait or disease outcome, and not just those reaching a pre-specified *P* -value threshold as shown in journal publications. It is this extensive availability of results that allows Gao *et al.* to relate genetic variants used as instrumental variables for adiposity traits to the cancer outcomes that they are interested in; the published GWAS for the cancer outcomes would not have reported on the adiposity variants unless they reached a pre-specified GWAS significance value.

## (One-sample) Mendelian randomization

**Figure 1.**
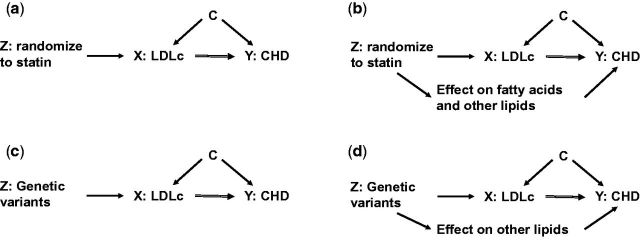
DAG of instrumental variable analyses in an RCT and MR study exploring the effect of LDLc on CHD. These are directed acyclic graphs (DAGs), thus the absence of an arrow between any two variables (nodes) indicates we do not consider it plausible that there is a causal effect between those two. Figure shows DAGs of instrumental variable (IV) analyses to test the causal effect of low-density lipoprotein cholesterol (LDLc) on CHD. In Figure 1 a and b, the IV is randomization to receiving a statin or not (i.e this is an example of IV analyses in an RCT). In Figure 1 c and d, the IV is genetic variants that are robustly related to LDLc (i.e. this is a Mendelian randomization study). Figure 1 a and c both illustrate the three key assumptions of IV analyses:

MR is a form of instrumental variable analysis that uses genetic variants as instrumental variables. It has been described as nature’s or your god’s randomized controlled trial (RCT), referring to the random allocation of genetic variants at conception that mean genetic variants are less likely to violate some of the assumptions of instrumental variable analyses than non-genetic instruments. ^5^^,^^6^ That analogy with RCTs is also useful when teaching MR to epidemiologists who feel anxious about genetics, as illustrated in Figure 1 which provides a revision of instrumental variable analyses and the key assumptions of this approach using an example of determining the causal effect of low-density lipoprotein cholesterol (LDLc) on coronary heart disease (CHD) via an RCT ( Figure 1 a and b) and via MR ( Figure 1 c and d). In both approaches the assumption that is most likely to be violated is the ‘exclusion restriction criteria’, ^7–9^ which states that the instrumental variable is only related to the outcome via its effect on the risk factor. Figure 1 also shows that, if the instrumental variable assumptions are correct, the estimate of the causal effect of the risk factor on outcome can be obtained from a simple ratio of the effect of the instrumental variable on outcome divided by the effect of the instrumental variable on risk factor. In the paper by Gao and colleagues, causal effect = log odds of cancer per allele of combined adiposity genetic variants ÷ regression coefficient of adiposity measure per allele of combined adiposity genetic variants.

i. that the IV ‘Z’ (randomization to statins in Figure 1 a and genetic variants related to LDLc in Figure 1 c) is (or is plausibly) causally related to the risk factor (LDLc in all figures);
ii. that confounding factors for the risk factor-outcome ‘X’-’Y’ association (here LDLc on CHD in all figures) are not related to the instrumental variable;iii. that the instrumental variable ‘Z’ only affects the outcome ‘Y’ (CHD) through its effect on the risk factor ‘X’ (LDLc).
In the RCT example we know that assumption (i) is true, and if the RCT is well conducted, then assumption (ii) will be true (other than chance associations). If, however, statins are directly (independently of LDLc) related to other factors which then affect CHD, assumption (iii) will be violated and the estimated causal effect will be a biased estimate of the true effect of LDLc. There is some evidence that statins do relate to a wide range of lipids and fatty acids in addition to LDLc, ^27^ though whether these are caused by the statins independent of LDLc and affect CHD is currently unknown. If they do (as shown in Figure 1 b) then the estimate of the LDLc effect on CHD is likely to be biased. In the MR example, selecting variants from large GWAS consortia where there is replication means that assumption (i) is likely to be correct. For assumption (ii) there is evidence that this is likely to be true. ^26^ As with the RCT example, in MR we are often most worried about assumption (iii) being violated through directional (horizontal) pleiotropy—i.e. the LDLc genetic variants influencing other factors independently of LDLc which in turn (independently of LDLc) affect CHD ( Figure 1 d). If the IV assumptions are correct (as illustrated in Figure 1 a and c) it can be seen that the magnitude of effect of LDLc on CHD can be easily calculated by the following *: e* ffect of LDLc on odds of CHD = log odds CHD on Z ÷ β LDLc on Z, where Z = randomization to statins (in the RCT example) or genetic variants for LDLc (in the MR example). For example, if in a well-conducted RCT randomization to a standard dose of statins reduces LDLc by 4 mmol/l and CHD by a relative reduction of 20% (odds ratio 0.80), then the causal effect of LCLc on CHD is a relative reduction of 5% (OR 0.95) per 1 mmol/l. It can also be seen that if assumption (iii) (the exclusion restriction criteria) is violated (as illustrated in Figure 1 b), then this estimate is biased as it is the combined effect of LDLc and any other lipids or fatty acids that are independently affected by statins and influence CHD.

## Two-sample Mendelian randomization

Two-sample MR exploits the fact that it is not necessary to obtain the effect of the instrumental variable-risk factor association (ratio denominator) and instrumental variable-outcome association (ratio numerator) from the same sample of participants. Indeed, there are some advantages to obtaining them from two different sets of participants. In particular ‘winners’ curse’, which can underestimate true causal effects in one-sample MR, ^10^ is unlikely to happen in two-sample MR, and unlike the impact of weak instrument bias in one-sample MR (which biases effects towards the confounded multivariable regression result), in two-sample MR weak instrument bias is towards the null. ^3^ The main advantage of using summary data from GWAS consortia in two-sample MR is the increased statistical power, particularly in relation to testing effects on binary disease outcomes. ^4^ The paper by Gao and colleagues illustrates this with the large numbers of cancer cases that they analyse: 15 748, 5100, 12 160, 4369 and 14 160, respectively, for breast, colorectal, lung, ovarian and prostate cancer. ^1^ The assumptions of two-sample MR are similar to those of one-sample MR, as are many of its strengths and limitations. It also has additional strengths and limitations in comparison with one-sample MR, which are summarized in Table 1 .

**Table 1.** Comparison of one-sample and two-sample MR

Assumption or other issue	One-sample MR	Two-sample MR
Instrumental variable is related to risk factor	• Can check this within the population with exposure and outcome (as both in same population) • Use F-statistic and R ^2^ of genetic instrument-risk factor association as measure of strength • Weak instrument biases towards the confounded regression analysis result	• Can check this within the population with exposure but need to be careful that the population used for testing genetic instrument-outcome association is the same as that testing instrument-risk factor (e.g. with respect to gender, sex, age, ethnicity etc.) • Use F-statistic and R ^2^ of genetic instrument-risk factor association as measure of strength • Weak instrument biases towards the null
Confounders of the risk factor-outcome association are not related to the genetic instrument	• Can (and should) check this for measured confounders	• If individual participant data are available for the two-samples can (and should) check this for measured confounders • When using summary data from publicly available GWAS results, will often not be possible to check this
Genetic instrument only related to the outcome through its effect on the risk factor	• Directional (horizontal) pleiotropy can be explored through use of different genetic instruments, multivariable instrumental variable analyses and MR-Egger ^8^^,^^9^	• Directional (horizontal) pleiotropy can be explored through use of different genetic instruments and MR-Egger ^9^ • In general. with summary data from large GWAS consortia, likely to have more power for these analyses which tend to be statistically inefficient
Subgroup analyses and effect moderation	• Possible if large sample sizes and data on the relevant stratifying risk factors (and genetic instruments for these) available	• Possible if individual participant data on the two samples and large sample sizes and data on the relevant stratifying risk factors (and genetic instruments for these) available • In general, with summary data from large GWAS consortia, it is unlikely to be able to test these
Bias from adjustments made in GWAS	• Not relevant as can decide within the one sample with genetic instrument, risk factor and outcome, what to adjust for.	• Not relevant if individual level data on both samples, as can then decide what to adjust for • If using summary data from published GWAS have to accept the adjustments that have been made in those GWAS, but should comment on the likely impact of this
Non-linear effects	• Methods available for testing this, though have additional assumptions and require large sample sizes ^24^^,^^25^	• Might be possible to apply the methods that have been developed for this, ^24^^,^^25^ if individual participant data available for the two samples • With summary data from large GWAS consortia, not clear how these methods could be applied currently.
‘Winner’s curse’	• If the same sample is used for GWAS discovery of the instrumental variables (i.e. effects on risk factor), with a *P* -value threshold to select variants (instruments), as the sample used for the testing of the instrument on outcome, the instrument-risk factor effect will be exaggerated and the instrument-outcome potentially underestimated. As a result the one-sample MR effect estimate will be an underestimate of the true causal effect ^10^	• Using two non-overlapping samples avoids this

## Overlapping samples and the use of summary or individual participant data

MR could be undertaken in one ‘sample’ of participants with genetic instrument and outcome data on all participants, and data on the risk factor in a (random) subsample. For example, UK Biobank will soon release GWAS data on all 500 000 participants and has already amassed large numbers of incident cases of cardiovascular disease and common cancers such as breast cancer. ^11^ Collection of unique imaging data on a subsample of 100 000 of those participants has begun, and thus MR to determine the causal effect of novel imaging biomarkers on common chronic disease outcomes, in which the genetic instrument-disease outcome association in 500 000 participants is divided by the genetic instrument-imaging biomarker association in the 100 000 subgroup, will soon be possible. However, this is a one-sample MR, as the subgroup ‘belong’ to the same study population. A one-sample MR study based on such a large sample would not have the advantages of two-sample MR, but it would have strong statistical power (including for testing causal effects on binary disease outcomes). In addition, it would have advantages from having individual participant data rather than summary data, though the very select nature of some large biobanks (the response rate for UK Biobank was less than 5%) might introduce additional biases.

The disadvantages of using summary data in two-sample MR are similar to those of meta-analysing summary data of RCTs or multivariable regression observational results—the quality of the pooled results is dependent on that of the individual studies. Thus, as the paper by Gao *et al.* illustrates (see below), one has to use the summary results presented, even when these are not idea, for example because they have been adjusted for co-variables that you would rather they had not been adjusted for or the sample used is not idea for your question.

When using summary GWAS data in what might be considered to be true two-sample MR, it is possible that the two samples overlap because of some cohort studies contributing to both GWAS (for example many adult cohort studies have contributed both to GWAS of adiposity measurements and also of disease outcomes such as CHD and type 2 diabetes). If this overlap is large, then some of the advantages of two-sample over one-sample MR are potentially lost, but the disadvantages of using summary data are maintained. Although it seems unlikely that this is an issue in the study undertaken by Gao and colleagues, methods to explore this ought to be included and their results discussed in any two-sample MR paper using summary data.

## Can we really use MR to test effects of adiposity on (breast) cancer at different life stages?

Gao and colleagues set out to explore ‘the potential causal relationship between obesity across different life stages and risk of multiple cancers’. Gao and colleagues examine the effects of childhood BMI and adult BMI, but they are not really able to determine effects at different life stages because of the correlation between BMI assessed at different ages and because of the nature of MR. Whereas one of the noted advantages of MR is that it generally assesses the cumulative effects of a risk factor over a long period of the life course (potentially from conception) without requiring repeat risk factor assessment and with little chance of regression dilution bias or reverse causation (confounding by prevalent disease), ^7^ this also brings a disadvantage in that MR is limited in the extent to which it can explore different life course models, such as whether exposure effects differ at different points in the life course. ^12^^,^^13^ From the original GWAS, 12 of the 15 child BMI overlapped with known adult BMI variants, ^14^ which illustrates the difficulty of distinguishing these two. Furthermore the MR-Egger test, ^9^ which the authors used to test violation of the exclusion restriction criteria, cannot be used to differentiate effects of adult from child BMI, as Gao and colleagues acknowledge. This is because MR-Egger is only valid if the effect of the genetic instrument on the risk factor of interest is independent of its effect on any other phenotypes that might violate that assumption. ^9^ In the case of childhood and adult BMI, we know that is unlikely to be the case.

The authors note that whereas their MR results suggest a protective effect of greater adult BMI on breast cancer, many observational studies have reported a protective effect of greater BMI on premenopausal breast cancer but a detrimental effect on postmenopausal breast cancer. They are unable with the summary data available to test differences in effect between pre- and postmenopausal breast cancer, as GWAS separated by these sub-phenotypes are not presented by the breast cancer consortia. However, one hypothesis regarding the positive association of BMI with postmenopausal breast cancer is that women who are fatter after the menopause are likely to have had a greater lifetime exposure to estrogen; but Gao and colleagues are able to examine effects with estrogen receptor-positive cancers and they find the same inverse association with these as seen for all breast cancer cases combined. ^1^ Furthermore, they note that their results are consistent with a recent one-sample MR study that found inverse associations of BMI with breast cancer in pre- and postmenopausal women, though at the time of writing this commentary that paper appears to be unpublished. The authors speculate that the protective effect of adult BMI on breast cancer (including postmenopausal) might represent a complex interplay between early life BMI and later weight gain. To test this using MR requires establishing different (independent) genetic variants related to early-life BMI and subsequent change in weight. Computationally that is difficult, but a recent GWAS of BMI trajectories from age 1 to 17 years shows some potential for future studies to be able to explore such possibilities. ^15^

## The provenance of adult BMI effects with cancers and other possible sources of bias in the conclusions for this study

Contrary to *IJE* author recommendations and recent guidance from the American Statistics Society, ^16^ Gao *et al.* largely base their conclusions on findings with a *P* -value equivalent to < 0.05 after multiple testing. Greater adult BMI, but not waist-hip ratio (WHR), is concluded to decrease breast cancer and increase ovarian, lung and colorectal cancer risk. However, some of the point estimates for BMI and WHR are not that dissimilar. Thus, it is concluded BMI reduces breast cancer risk {odds ratio[OR] 0.66 [95% confidence interval (CI): 0.57, 0.77)]}, but the same is not concluded for WHR [0.73 (0.53, 1.00)]. Similarly, an odds ratio of 1.27 (1.09, 1.49) for the effect of adult BMI on all lung cancers is declared as a positive result but the same conclusion is not made for an odds ratio of 1.33 (95%CI: 0.75, 2.36) for the MR effect of WHR on squamous lung cancer.

A related issue is whether the WHR findings could have been biased towards the null more than BMI findings. One disadvantage of using summary data is that you have to take the results as analysed in the original study. The WHR variants used by Gao and colleagues were adjusted for BMI, which the authors do not seem to acknowledge. This adjustment is likely to have biased the effect of genome-wide variants associated with unadjusted WHR (away from the null). ^17^ Thus, the MR estimate of the effect of unadjusted WHR on cancer would be to bias it towards the null because the denominator of the ratio (the genetic instrument-WHR association) will be exaggerated due to adjustment for BMI.

A further potential explanation for why most of the emphasized (based on statistical testing) MR results are seen for adult BMI, rather than any of the other adiposity risk factors, is that the genetic instrument for adult BMI is stronger than for the other traits. Weak instrument bias in one-sample MR results in bias towards the confounded multivariable regression result, but in two-sample MR the bias is towards the null ( Table 1 ). ^3^ Gao and colleagues do not provide any information on the strength of the different instrumental variables, such as the F-statistic or R ^2^ for the genetic instrument-adiposity trait associations. From the original papers it can be seen that the instrument for adult BMI is stronger than for the other traits (the respective R ^2^ for adult BMI, birthweight, child BMI and WHR, are: 0.027, 0.008, 0.020 and 0.013; the R ^2^ for child BMI is only for the three novel SNPs but, as the authors of the original paper point out, it was calculated on a relatively small sample and needs to be treated with some caution). ^14^^,^^18–20^ Interestingly, although Gao *et al.* use the most up-to-date BMI GWAS data, ^20^ they do not do the same for WHR, despite the most recent GWAS for WHR adjusted for BMI identifying 33 additional variants (as well as confirming the 14 used here from the earlier GWAS) and being published around the same time as the most up-to-date BMI GWAS. ^21^

For two-sample MR to be valid, the two samples have to be from the same underlying population, but for the sex-specific cancers in the paper by Gao *et al.* , this does not seem to be the case. According to data presented by Gao in their Supplementary Table 1, it seems that the association of the genetic instrumental variable with each adiposity trait has been taken from samples that combine females and males, whereas for the association of the genetic instrument with breast and ovarian cancer, females only are included and with prostate cancer males only are included. ^1^ This assumes that the genetic variants do not differ between women and men in their relationship to the adiposity risk factors. This issue is not discussed by Gao *et al.*

I looked at the four original GWAS papers to explore whether there was any difference in the GWAS of the adiposity traits by sex. ^14^^,^^18–20^ For birthweight and child BMI, there seemed to have been no attempt to explore sex differences, which likely reflects the low power in those studies to do that. Thus, it is impossible to know whether the assumption of no sex differences holds for these two risk factors. For adult BMI, sex differences were reported and marked differences were found for two of the 77 variants (stronger associations in women compared with men). Differences in just two of the 77 variants might not have been sufficient to bias the results for adult BMI with the sex-specific outcomes, but it is disappointing that the authors did not use the sex-specific beta values for each variant with the sex-specific outcome nor clarified in the paper that the denominators combined data from both sexes.

What is more surprising is that they seem to have also used sex-combined results for determining effects of WHR adjusted for BMI, despite the fact that it is clear from the title of the original GWAS paper that sex differences were examined and found ^19^ ( Table 2 ). Seven of the 14 WHR adjusted for BMI variants used by Gao and colleagues were stronger in females compared with males ( Table 2 ), with 19 of the 44 variants in the more up-to-date GWAS being stronger in females (and one stronger in males). ^21^ In both GWAS, the results of the per allele effect of the genetic instrument on WHR adjusted for BMI is notably stronger in females than males. As this is the denominator of the MR ratio estimate, it means that the estimated effect of WHR adjusted for BMI for female cancers (breast and ovarian) may be exaggerated and those for prostate cancers underestimated.

**Table 2.** Per allele effect magnitude of GWAS significant SNPs with waist-hip ratio (adjusted for body mass index) by sex from the original GWAS and used in two-sample MR of cancer effects by Gao and colleagues

SNP rs number	GWAS effect in women	GWAS effect in men	GWAS effect in women and men combined	Effect magnitude used in Gao *et al.*
Rs9491696	0.050*	0.031	0.042	0.042
Rs6905288	0.052*	0.020	0.036	0.036
Rs984222	0.034	0.035	0.034	0.034
Rs1055144	0.044	0.035	0.040	0.040
Rs10195252	0.054*	0.010	0.033	0.033
Rs4846567	0.059*	0.005	0.034	0.034
Rs1011731	0.029	0.028	0.028	0.028
Rs718314	0.042*	0.017	0.030	0.030
Rs1294421	0.031	0.025	0.028	0.028
Rs1443512	0.040*	0.018	0.031	0.031
Rs6795335	0.038*	0.011	0.025	0.025
Rs4823006	0.030	0.015	0.023	0.023
Rs6784615	0.047	0.039	0.043	0.043
Rs681681	0.024	0.019	0.022	0.022

All values are the per allele difference in waist-hip ratio (WHR) adjusted for body mass index (BMI). In the GWAS there was strong statistical evidence that each association had a low probability of being due to chance, particularly in women ( *P*_women only_ 1.55 × 10 ^−6^ to 3.84 × 10 ^−34^ ; *P*_men only_ 0.043 to 9.41 × 10 ^−13^ ; *P*_combined_ 1.9 × 10 ^−9^ to 1.8 × 10 ^−40^ ). Gao *et al.* appear to have generated an allele score of the effects from the sex combined results in all of their analyses, including those with sex-specific outcomes (breast, ovarian and prostate cancer). All variants combined explained 1.34% and 0.46% of the variation in WHR adjusted for BMI in women and men, respectively. The combined per-allele effect in women was stronger than in men, specifically; for those marked with an asterisk (*), there was strong statistical evidence of a sex difference ( *P*_sex difference_ 1.9 × 10 ^−3^ to 1.2 × 10 ^−13^ )

The extent to which bias towards the null as a possible result of weak instrument bias and adjustment of WHR for BMI (discussed above) is balanced by possible exaggeration of the true effect as a result of not using sex-specific data for the genetic instrument-WHR association in the female cancers, is impossible to tell. But this study does illustrate some of the pitfalls of using summary GWAS data and methods that might be used to limit these. Thus, I would suggest the following recommendations for using summary data in two-sample MR.

- Ensure that the two samples are from the same populations. If this is not the case (as in this paper for the sex-specific cancers), check the original paper publications and/or contact the original authors to see if it is possible to obtain results from the same population (here sex-specific results). If that is not possible, consider possible biases, undertake sensitivity analyses and/or consider whether it is appropriate to undertake the analyses.
- Report on the extent of any overlap between the two samples. This will require searching of the original publications and/or the consortia website. If overlap is large, then the study should be considered to be more like a one-sample MR and the discussion of strengths and limitations should be directed towards those of one-sample MR.
- Determine whether any covariables have been adjusted for in the original GWAS and report on this. If there have been adjustments, ensure that presentation and interpretation of results take this into account.
- Report how risk factors and outcomes were assessed, including whether disease cases were prevalent, incident or a mixture. Consider whether measurement error and/or survivor bias (where predominantly prevalent cases are used) might have influenced findings.
- Describe any key additional analyses that would have been important to conduct, such as of sub-phenotypes or interactions, that were not possible because of the summary data.

## Beyond Mendelian randomization—what can we learn from genetic epidemiology?

What strikes me in watching (and participating in) the development of GWAS and MR over the past decade is how slow those of us largely working in epidemiology, including in intervention research, have been to do what we all know is good science. Our genetic colleagues have led the way in ensuring replication in large collaborations where ‘team science’ is appreciated and for the large part appropriately rewarded. Those developing MR as a method have from the start been very open about its limitations and have worked at developing methods to test and limit sources of bias. ^2^^,^^3^^,^^9^ It is notable, for example, that Gao *et al.* comment on the ‘strong’ assumptions of MR, but rarely do we see such statements about the equally strong, and untestable, assumptions of conventional multivariable regression analyses. Now genetic epidemiologists have shown us how to provide complete open-access summary data, and it is likely that over the coming decade important and impactful use will be made of these data. ^4^

A decade before the first paper proposing the use of MR, ^2^ Lau and colleagues demonstrated that, had a cumulative meta-analysis been regularly updated, the beneficial effect of streptokinase in patients who had experienced an acute myocardial infarction would have been established by 1973, with the further randomization of over 35 000 patients after that date simply confirming the original results and delaying widespread implementation of a life-saving treatment. ^22^ In 1997, Egger and Davey Smith showed the same with respect to beta-blockers and mortality after acute myocardial infarction. ^23^ Those retrospective findings could have been identified prospectively with easy open access to complete summary data of everything tested in all RCTs. However, we still lack such access.
